# Supplementary material for: Growth of ocean thermal energy conversion resources under greenhouse warming regulated by oceanic eddies
Source: Nat Commun. 2022 Nov 25;13:7249. doi: 10.1038/s41467-022-34835-z (PMC9700850; doi:10.1038/s41467-022-34835-z)
Supplement: Supplementary file 1 — Supplementary Information [file 41467_2022_34835_MOESM1_ESM.pdf]

Supplementary Information for

Growth of Ocean Thermal Energy Conversion Resources under Greenhouse Warming Regulated by Oceanic Eddies

Authors: Tianshi Du[1,2], Zhao Jing[1,2*], Lixin Wu[1,2], Hong Wang[1,2], Zhaohui Chen[1,2], Xiaohui Ma[1,2], Bolan Gan[1,2] and Haiyuan Yang[1,2]

Affiliations:

[1]Frontiers Science Center for Deep Ocean Multispheres and Earth System and Key Laboratory of Physical Oceanography, Ocean University of China; Qingdao, China.

[2]Laoshan Laboratory, Qingdao, China.

*Corresponding author. Email: jingzhao@ouc.edu.cn

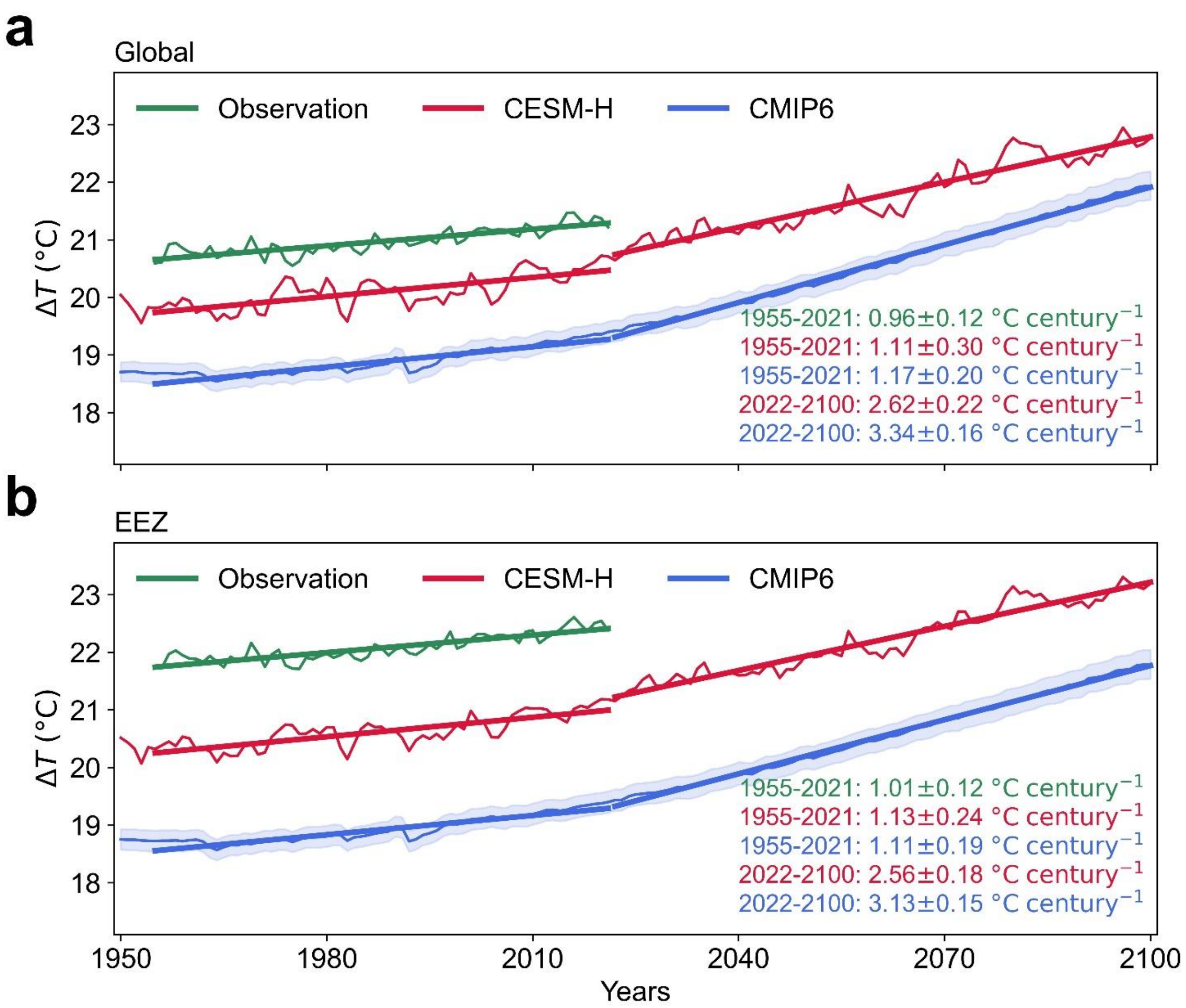

Supplementary Fig. 1 | Ocean thermal stratification in the observation and climate model simulations. **a**, Horizontal mean ocean thermal stratification ΔT within the ocean thermal energy conversion (OTEC) region derived from the observation (green), high-resolution Community Earth System Model (CESM-H) (red), and ensemble mean of the coupled global climate models (CGCMs) in the Coupled Model Intercomparison Project Phase 6 (CMIP6) (blue). The shading corresponds to the standard error of the CMIP6 CGCM ensemble mean. The numbers on the bottom right corner show the slope of linear trends along with its standard error. Note that the OTEC regions differ among datasets and vary with time. To eliminate these variabilities, the horizontal average is always performed over the union of all the instantaneous OTEC regions during 1992-2021 in the observation. **b**, Same as **a**, but for ΔT over the OTEC region within exclusive economic zone (EEZ).

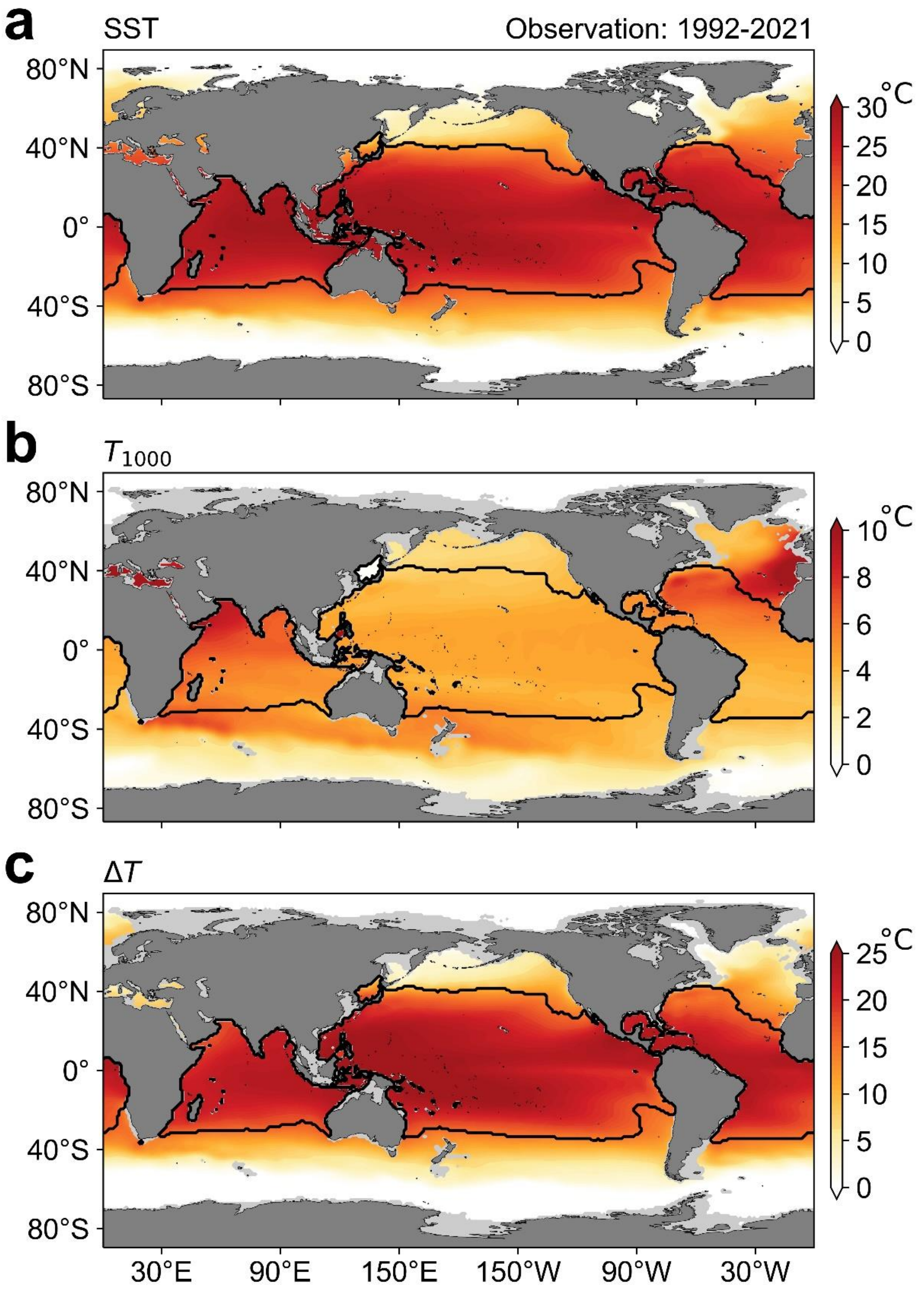

Supplementary Fig. 2 | Spatial distribution of time-mean sea surface temperature (SST) (a), deep ocean temperature at 1000 m T_{1000} (b), and their difference ΔT (c) during 1992-2021 in the observation. The black solid line denotes the zero contour of the time-mean P_{net} during 1992-2021 in the observation.

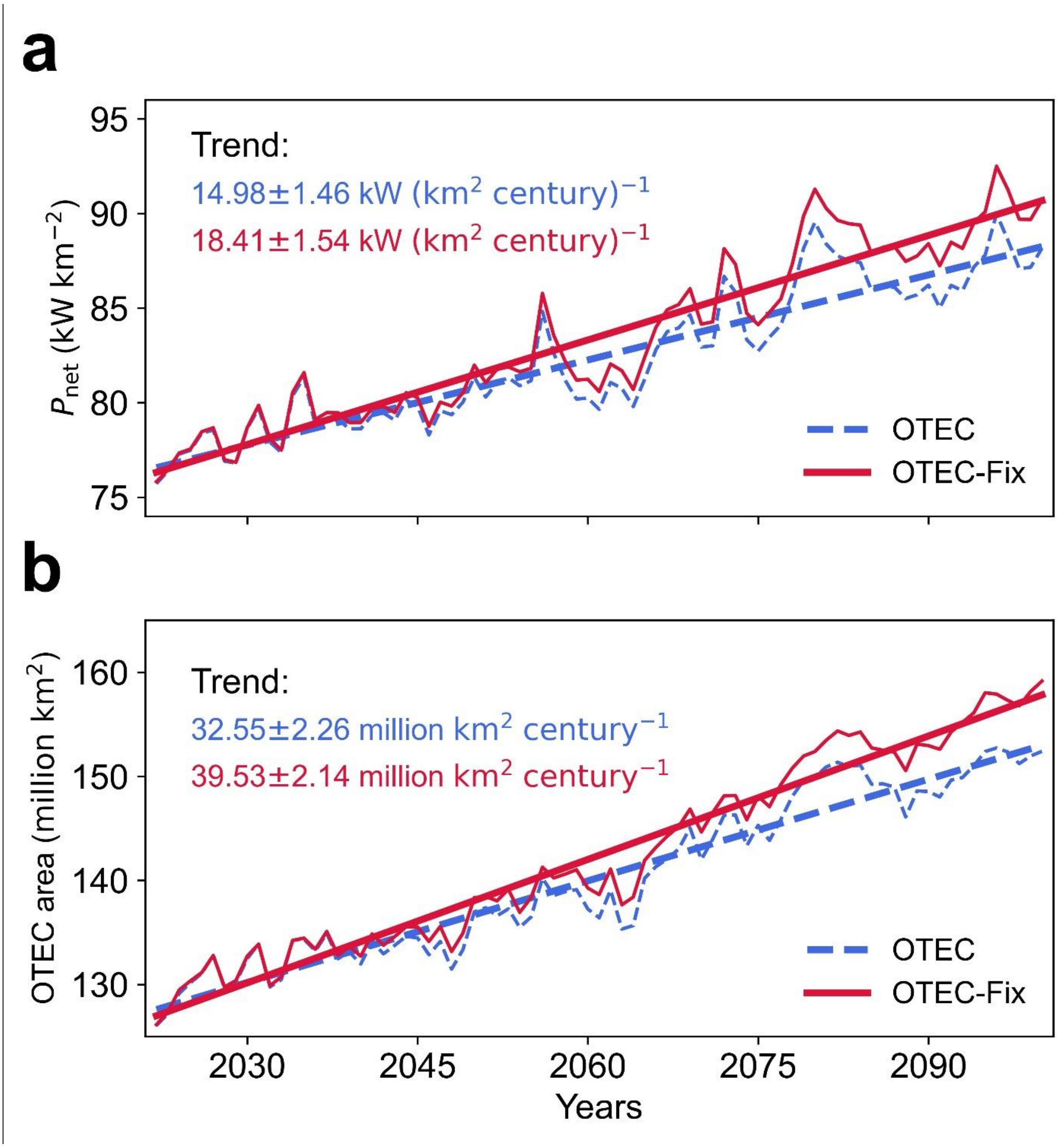

Supplementary Fig. 3 | Offsetting effect of deep ocean warming on the increases of ocean thermal energy conversion (OTEC) power potential density and area of OTEC region under the high carbon emission scenario derived from the high-resolution Community Earth System Model (CESM-H). a, Time series of global mean P_{net} and $P_{\text{net}}^{\text{fix}}$ during 2022-2100. The numbers on the top left corner show the slope of their linear trends along with its standard error. **b**, Same as **a,** but for the area of OTEC region.

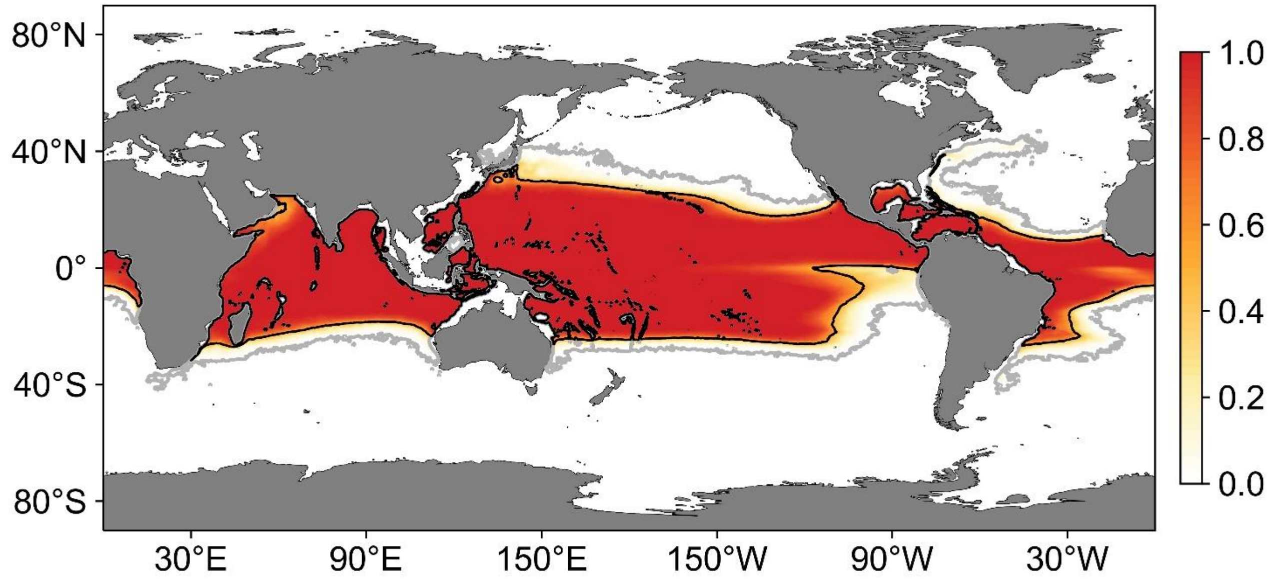

Supplementary Fig. 4 | Frequency of temperature difference (Δ*T*) exceeding the 20 °C threshold during 1992-2021 in the high-resolution Community Earth System Model (CESM-H). Grey and black lines denote the contours of 0 and 0.5, respectively. The domain between the two contours is defined as the margin of the ocean thermal energy conversion (OTEC) region during 1992-2021.

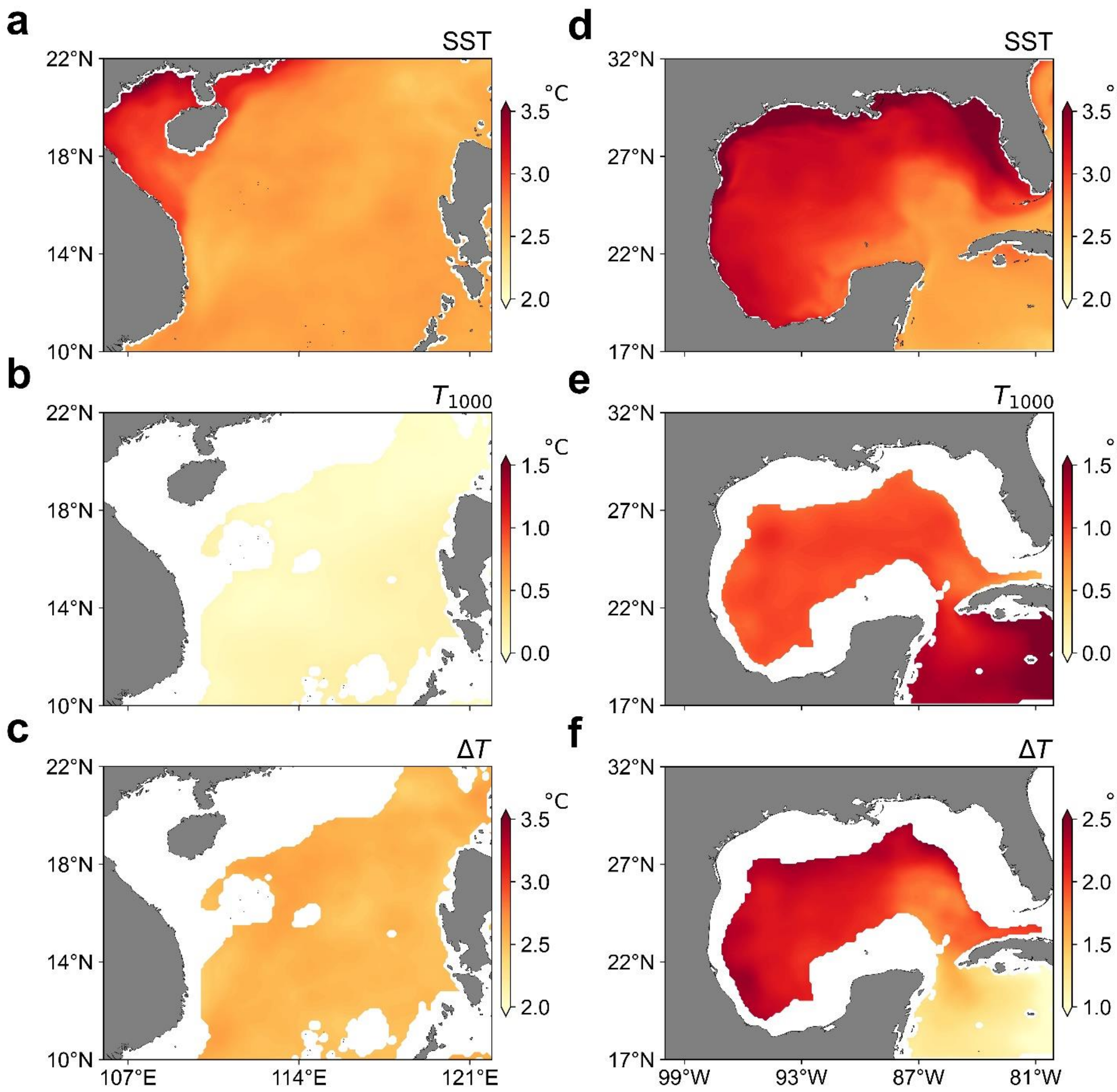

Supplementary Fig. 5 | High-resolution Community Earth System Model (CESM-H) simulated spatial distribution of time-mean sea surface temperature (SST) (**a**), deep ocean temperature at 1000 m T_{1000} (**b**), and their difference ΔT (**c**) in the South China Sea during 2071-2100 minus their counterparts during 1992-2021. **d-f**, same as **a-c**, but in the Gulf of Mexico.

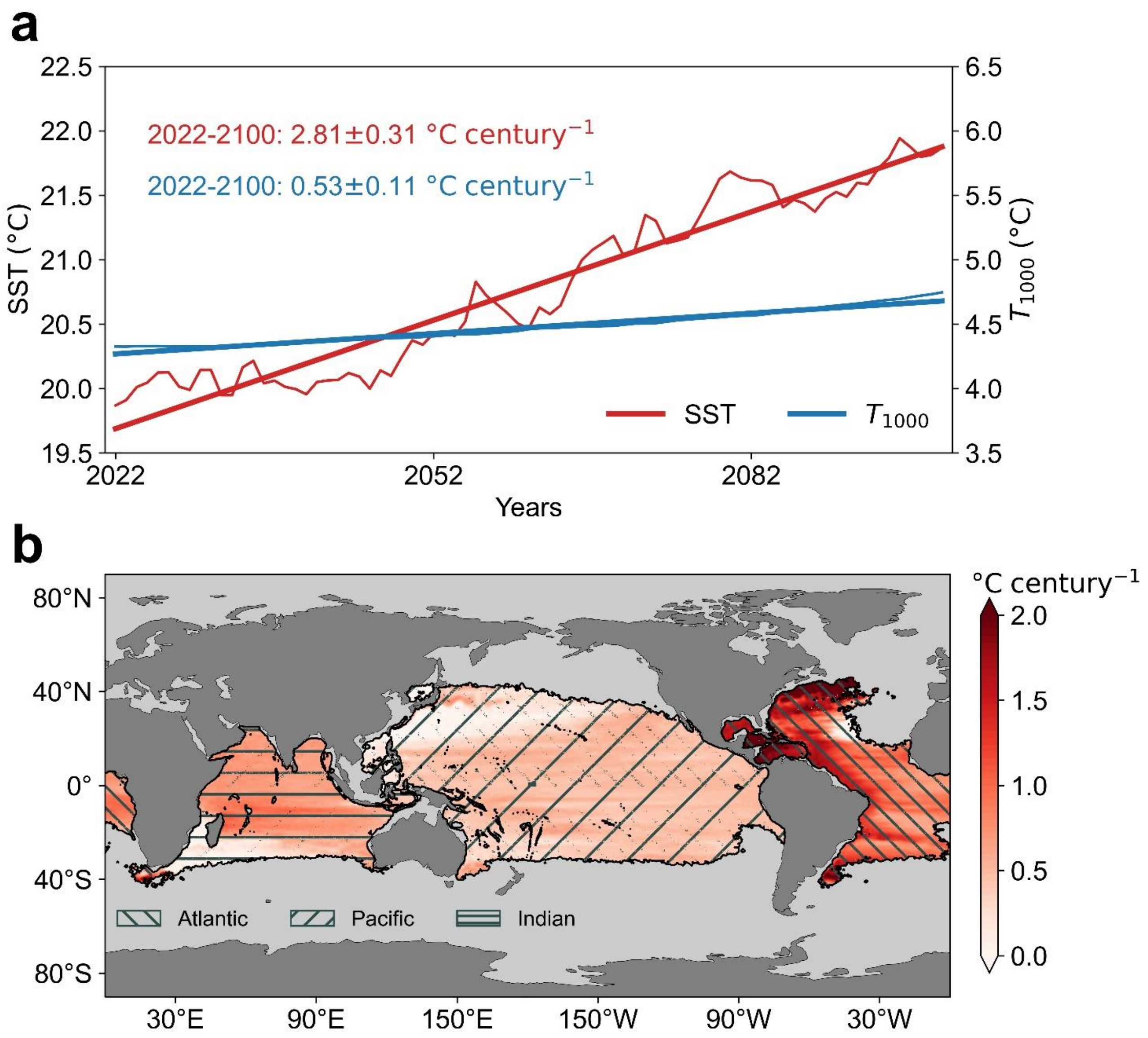

Supplementary Fig. 6 | Projected future trends of ocean temperature under greenhouse warming by the high-resolution Community Earth System Model (CESM-H). a, Time series of global mean sea surface temperature (SST) and temperature at 1000 m during 2022-2100 projected by the CESM-H. The numbers on the top left corner show the slope of linear trends along with its standard error. **b**, Geographic distribution of the linear trend of temperature at 1000 m during 2022-2100 projected by the CESM-H. The black line is the zero contour of the time-mean P_{net} from 2071 to 2100, encompassing the ocean thermal energy conversion (OTEC) region during that period. The OTEC regions in the Pacific, Atlantic and Indian Oceans are labelled with different hatching patterns.

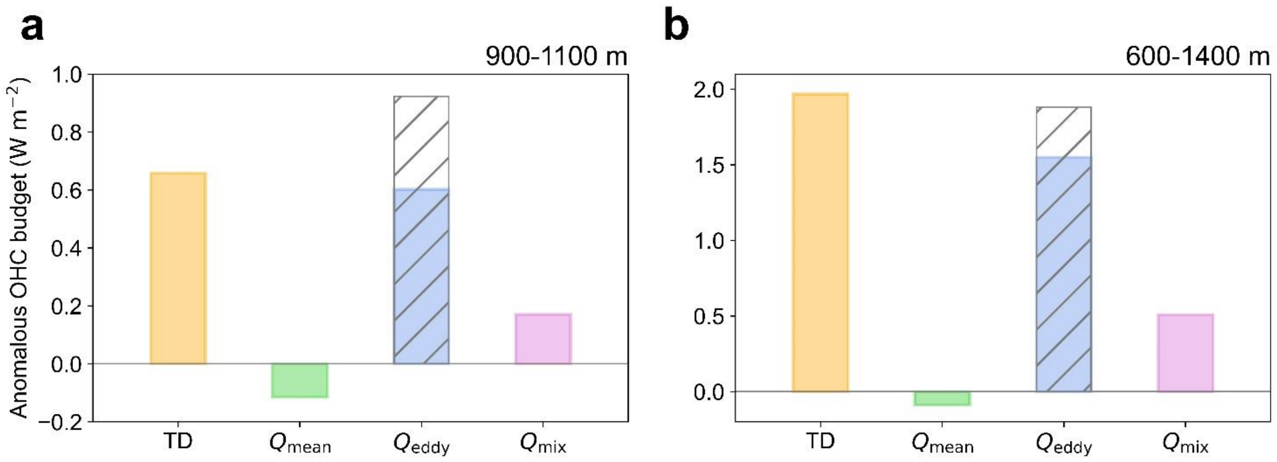

Supplementary Fig. 7 | Same as Fig. 4b but for the 900-1100 m (**a**) and 600-1400 m (**b**) water column. The contribution of vertical mesoscale eddy heat transport at 900 m and 600 m to the heat transport convergence by mesoscale eddies (Q_{eddy}) is marked by the hatched lines in (**a**) and (**b**), respectively.

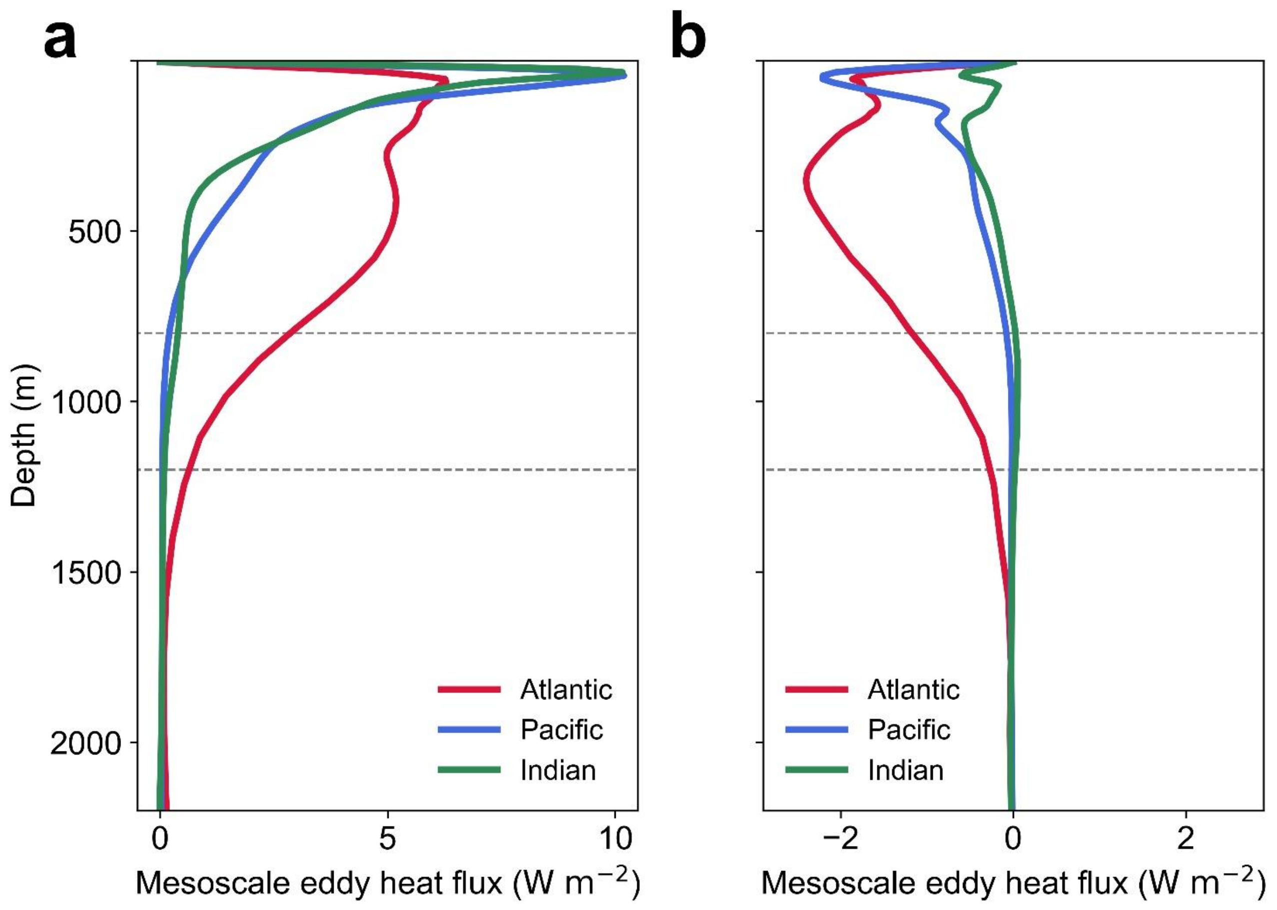

Supplementary Fig. 8 | Horizontally averaged mesoscale vertical eddy heat transport in the ocean thermal energy conversion (OTEC) region of Atlantic, Indian, and Pacific Oceans derived from the high-resolution Community Earth System Model (CESM-H). a, The time-mean value during 1992-2021, and **b**, its difference under greenhouse warming (2071-2100 minus 1992-2021). The gray dashed lines represent the depths of 800 and 1200 m.

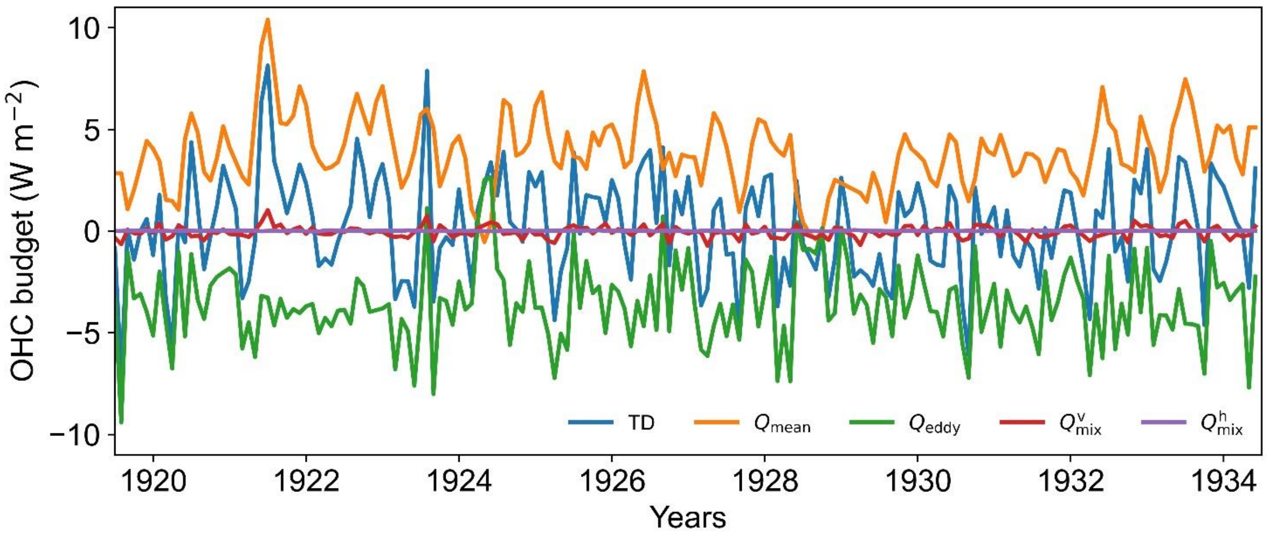

Supplementary Fig. 9 | Time series of individual terms in the ocean heat content (OHC) budget for the 800-1200 m water column averaged over the Atlantic ocean thermal energy conversion (OTEC) region during 1920-1934. The high-resolution Community Earth System Model (CESM-H) saves the entire diagnostic output for the temperature governing equation during 1920-1934, in which case the OHC tendecy (TD), heat transport convergence by mean flows Q_{mean}, heat transport convergence by mesoscale eddies Q_{eddy}, vertical mixing Q^{v}_{mix} and horizontal mixing Q^{h}_{mix} can be explicitly computed.

Supplementary Table 1 | A list of coupled global climate models (CGCMs) in the Coupled Model Intercomparison Project Phase 6 (CMIP6) used in this study.

CGCM	Nominal Resolution (km)
ACCESS-CM2	250
ACCESS-ESM1-5	250
BCC-CSM2-MR	100
CAMS-CSM1-0	100
CAS-ESM2-0	100
CESM2	100
CESM2-WACCM	100
CIESM	100
CMCC-CM2-SR5	100
CMCC-ESM2	100
CNRM-CM6-1	100
CNRM-ESM2-1	100
CanESM5	100
CanESM5-CanOE	100
E3SM-1-1	100
EC-Earth3	100
EC-Earth3-Veg	100
EC-Earth3-Veg-LR	100
FGOALS-f3-L	100
FGOALS-g3	100
FIO-ESM-2-0	100
GFDL-ESM4	50
GISS-E2-1-G	250
HadGEM3-GC31-LL	100
INM-CM4-8	100
INM-CM5-0	100
KIOST-ESM	100
MCM-UA-1-0	250
MIROC-ES2L	100
MIROC6	100
MPI-ESM1-2-HR	50
MPI-ESM1-2-LR	250
MRI-ESM2-0	100
NESM3	100
TaiESM1	100
UKESM1-0-LL	100
